# Cardiac arrhythmias and electrolyte disturbances in colic horses

**DOI:** 10.1186/s13028-014-0058-y

**Published:** 2014-10-02

**Authors:** Eva Z Hesselkilde, Mette E Almind, Jesper Petersen, Mette Flethøj, Kirstine F Præstegaard, Rikke Buhl

**Affiliations:** Department of Large Animal Sciences, Faculty of Health and Medical Sciences, University of Copenhagen, Højbakkegård Allé 5, DK-2630 Taastrup, Denmark

**Keywords:** Equine, Colic, ECG, Arrhythmias, Heart rate, Electrolytes

## Abstract

**Background:**

Despite increased focus on cardiac arrhythmias in horses, the nature and prevalence is still poorly described. Case reports suggest that arrhythmias occurring secondary to systemic disease are seen more commonly in the clinic than arrhythmias caused by cardiac disease. The aim of this study was to investigate the prevalence of arrhythmias in colic horses referred for hospital treatment. Associations between electrolyte disturbances and arrhythmias were also investigated.

The study population consisted of eight control horses and 22 referred colic horses. A Holter electrocardiography (ECG) was recorded during the first 24 hours of admission. The ECG’s were analysed by a software program followed by manual visual inspection. Arrhythmias registered included second degree atrioventricular (AV) blocks, supraventricular premature complexes (SVPCs), and ventricular premature complexes (VPCs). Blood was collected at admission and again between 12 and 24 hours after ECG was applied, and analysed for concentrations of potassium, sodium, ionised calcium, chloride, glucose, and L-lactate.

**Results:**

Heart rate was 37.4 ± 3.7 bpm in the control group, and 51.6 ± 11.8 bpm, in the colic group, which was significantly different (*P* < 0.0001). AV blocks and SVPCs were found in both groups, however only colic horses showed VPCs. No significant difference between the two groups was found for AV blocks, SVPCs, and VPCs (P = 0.08 - 0.76). The mean levels of potassium, sodium, ionized calcium, and chloride were significantly lower in the colic group compared to the control group at admission. Mean levels of glucose and L-lactate were significantly elevated in the colic group (P < 0.05).

**Conclusions:**

This study describes prevalence of cardiac arrhythmias and electrolytes concentrations in colic horses compared to healthy controls. Although we only observed VPCs in the colic horses, no significant differences between colic horses and controls were found. Despite the colic horses having electrolyte changes at admission no correlation was found between the electrolyte disturbances and cardiac arrhythmias. Although no clear conclusions can be drawn from the present study, the results indicate that relatively mild colic per se is not pro-arrhythmogenic, whereas severe colic probably are more likely to result in ventricular arrhythmia.

## Background

In recent years, focus has increased on prevalence and causes of cardiac arrhythmias in horses both in performance horses, diseased horses [1-5], and during and after anaesthesia [6,7]. Despite limited data describing the prevalence of cardiac arrhythmias, case reports support that arrhythmias occur secondary to systemic diseases [4,8]. Specifically, arrhythmias seem to occur more frequently with gastrointestinal (GI) disease than with primary myocardial disease [4,8]. Possible causes of arrhythmias secondary to GI disease include the direct effects of endotoxins on the myocardium, autonomic imbalance resulting from GI distension, metabolic, electrolyte, or acid-base imbalances [4]. Specific electrolytes that are more frequently associated with arrhythmias in small animals and horses include potassium, calcium and magnesium [9-11]. For the clinician, it is currently a challenge to evaluate the significance of cardiac arrhythmias in the often very painful and systemically affected colic horse, since the nature and prevalence of arrhythmias in these patients are based primarily on case reports [4,8].

The objective of the present study was therefore to compare the prevalence of cardiac arrhythmias in horses referred to the hospital for colic with healthy control horses. Secondary, we wished to determine differences in electrolyte levels between colic horses and healthy horses and to evaluate potential associations between arrhythmias and electrolytes disturbances.

We hypothesised that horses suffering from colic would exhibit a higher prevalence of arrhythmias and more severe arrhythmias during the first 24 hours after admission to the hospital than control horses. Also, we hypothesised that deficits in potassium, calcium, and/or magnesium, and increased levels of glucose or lactate were associated with cardiac arrhythmias.

## Methods

### Study population

Two groups of horses were included in the present study, and data were obtained from February to April 2010. The control group consisted of 8 clinical healthy mares, mean age 12.7 years (range 7-30 years) stabled at the University of Copenhagen and used for teaching purposes. The case group consisted of 22 horses of different breeds, with a mean age of 10.0 years (range 2-22 years) including 12 mares, 9 geldings, and 1 stallion. All horses were referred due to colic to the Large Animal Teaching Hospital, University of Copenhagen. Horses were only included, if two of the authors were present at the hospital to mount the horse with the electrocardiographic (ECG) equipment when admitted. The inclusion criteria was colic prior to hospitalization and that the horses were not taken to surgery. Clinical examination of the case group was performed by the hospital veterinarians. The authors performed clinical examinations of the control horses to ensure that they were clinically healthy. The procedures performed in the colic horses were all a part of the standard procedures performed in the hospital. Therefore no permission was required from the Danish Animal Experimentation Inspectorate. However, the studies performed in the control horses were approved by The Danish Animal Experiments Inspectorate (license number 1010-561-1856).

### ECG analysis

The control horses had ECG recorded for 24 hours while stabled in loose boxes. Four horses were measured at a time, and during measurements they were stabled together in separate boxes. One horse had only 16 hours registered due to electrode detachment. For colic horses, ECG equipment was applied within one hour after admission, and ECG was recorded for up to 24 hours, mean 15 hours (range 1-24 hours). Self-adhesive ECG electrodes were attached on saddle girth area as previously described [3]. Electrodes were covered by an elastic girth. A leather girth equipped with a safety pocket for the ECG unit was applied over the elastic girth. For all horses, a modified base-apex lead ECG was recorded (Krutech Televet 100, Kruuse A/S, Denmark) and monitored telemetrically on a laptop. Data was stored on a SD card (2 GB, Transcend) for later analysis.

The ECG recordings were subjected to a rhythm analysis by the integrated software program (Televet 100® version 5.0, Kruuse A/S, Denmark). The software recognizes R-waves and thereby calculates RR-intervals and heart rates. The maximum accepted deviation in RR-interval was set to 20% according to a previous study [2]. The RR-interval deviations were manually controlled to ensure that the software has marked the R-waves correctly, and an additional complete manual read-through of the ECGs was performed to check the morphology of QRS complexes. Arrhythmias were classified manually by one observer (RB) based on the following arrhythmia definitions:

- Second degree AV block: P wave not associated with any following QRS complex, and double length of RR-interval [12].
- SA Block: Interval twice the normal RR-interval with no P-wave or QRS complex occurring [12].
- Supraventricular premature complex (SVPC): RR-interval decreased more than 20% in duration from the previous RR-interval, and no change in configuration of the QRS-complex. For the premature beat, the P wave may not have been visible if HR was high, but most often it could be identified in the preceding T wave. As long as the QRS-complex had similar morphology as the preceding complexes it was considered a SVPC. The rhythm should be regular before and after not to misinterpret it as sinus arrhythmia [2].
- Ventricular premature complex (VPC): RR-interval decreased more than 20% in duration from the previous RR-interval, and QRS complex of abnormal morphology compared to the previous normal QRS complex. No P wave identified before the premature complex. If three or more consecutive VPCs were observed this was classified as ventricular tachycardia (VT) [2,8].

Sinus arrhythmia was not recorded in the present study, but the SVPCs were carefully evaluated to ensure that the premature beat was not part of sinus arrhythmia. Due to variance in recording time, number of arrhythmias was divided by the number of recorded hours for each horse giving a mean number of arrhythmias per hour.

### Heart rate

RR intervals and associated time points were exported to spread sheets (Microsoft Excel 2010) by the Televet software (Televet 100® version 5.0). Mean, standard deviation (SD), and range (minimum and maximum) were calculated for each horse based on the entire ECG period.

### Electrolyte analysis

Venous blood samples were collected from the jugular vein in heparinised 3 mL syringes. In the colic group this was executed at admission (T = 0), whereas in the control group the samples were collected at initiation of ECG (T = 0). All blood samples were analysed using a blood gas analyser (Radiometer ABL^TM^725, Radiometer) within 10 minutes of sampling. Concentrations of potassium (K^+^), sodium (Na^+^), ionised calcium (iCa^2+^), chloride (Cl^-^), glucose, L-lactate, and pH were measured. Packed cell volume (PCV) was measured manually for all horses by centrifuging heparinized blood in a capillary tube. For the colic horses, an additional blood sample was obtained at admission (T = 0). This blood sample was collected in an 8.5 mL serum separator tube to measure total magnesium (tMg^2+^) (ADVIA® 1800 Chemistry System, Siemens). From the 8 horses in the control group and 14 of the horses in the colic group, a second venous blood sample was collected from the jugular vein in a heparinised 3 mL syringe in the period from 12 to 24 hours after initiation of ECG registration (T = 24). This blood sample was analysed using the blood gas analyser (Radiometer ABL^TM^725, Radiometer) within 10 minutes as for T = 0. The remaining 8 horses from the colic group were either discharged (2 horses) or euthanized (6 horses) within the 24 hours and therefore no second blood sample was obtained. Due to economical restrictions in the study, total magnesium was neither measured in the control horses nor at T = 24 in the colic group.

### Statistical analysis

Statistical analyses were carried out using Prism 5.04 (Prism, GraphPad). Arrhythmias were measured as the number of AV blocks, SVPCs and VPCs, each divided by hours of measured ECG. Association between the number of arrhythmias/hour as a function of electrolyte concentrations at T = 0 was tested for all horses (n = 30) by linear regression. Changes in means within groups from T = 0 to T = 24 in potassium, sodium, ionised calcium, chloride, glucose, and L-lactate for the control and colic horses were evaluated using paired *t*-test. Distributions of electrolytes, metabolites, and arrhythmias were compared for each variable between the colic group and the controls by an unpaired *t*-test with Welch’s correction. Electrolyte variables were the concentration of potassium, sodium, ionised calcium and chloride. Metabolites included concentrations of glucose and L-lactate. A Mann-Whitney test or a Wilcoxon matched pair test was used for data that did not pass a normality test (D’Agostino&Pearson omnibus normality test). All data is presented as mean ± SD and a 5% significance level was used. Asterisks indicate; * = *P <* 0.05*,* ** = *P <* 0.01*,* *** = *P <* 0.001.

## Results

Of 22 referred colic horses, 12 horses were diagnosed with large colon impaction, three with displacement of the colon, three with nephrosplenic entrapment of the large colon (two of these had concurrent colon impaction), three with peritonitis, and in three horses no apparent diagnosis could be confirmed (Table 1). None of the horses were taken to abdominal surgery. Eight of the horses were euthanized within the first 24 hours, of which five were euthanized due to economic restrictions by the owner, and for the remaining three the veterinarian in charge did not advise surgery due to non-surgical conditions and poor prognosis.

**Table 1:** **Descriptive analysis of electrocardiographic results and heart rate in control (n = 8) and colic (n = 22) horses**

**Horse**	**Diagnose**	**Survival**	**ECC recording (hours)**	**HR Mean (range)**	**AV/SA block**	**SVPC**	**VPC**
**Control 1**	-	1	24	40 (22-150)	85	33	0
**Control 2**	-	1	24	43 (30-122)	2	4	0
**Control 3**	-	1	24	35 (25-93)	0	10	0
**Control 4**	-	1	24	40 (24-107)	5	66	0
**Control 5**	-	1	24	38 (27-111)	3	12	0
**Control 6**	-	1	16	36 (28-69)	400	1	0
**Control 7**	-	1	24	36 (27-135)	2	24	0
**Control 8**	-	1	24	31 (21-135)	3	34	0
**Colic_1**	Colonic impaction	1	24	40 (27-104)	3	5	0
**Colic_2**	Unspecific	1	24	56 (24-162)	0	31	0
**Colic_3**	Retroflexion of colon	0*	14	67 (25-125)	7	210	0
**Colic_4**	Nephrosplenic Entrapment	1	24	61 (33-135)	6	7	2
**Colic_5**	Peritonitis and necrotic typhlocolitis	0*	10	68 (39-133)	0	43	22**
**Colic_6**	Duodenal hypertrophy rupture of diverticle and peritonitis	0*	9	63 (32-87)	2	1	0
**Colic_7**	Colonic impaction	1	18	41 (30-178)	112	42	0
**Colic_8**	Unspecific	1	24	62 (29-138)	0	20	0
**Colic_9**	Acute colonic torsion	0*	4	41 (25-89)	0	43	3
**Colic_10**	Colonic impaction	1	24	39 (23-157)	3	12	0
**Colic_11**	Local peritonitis and ventricular ulceration	0*	1	53 (46-71)	0	1	0
**Colic_12**	Colonic impaction	1	24	46 (24-166)	90	25	0
**Colic_13**	Colonic impaction	1	9	54 (35-140)	0	12	0
**Colic_14**	Displacement of ascending colon	0*	1	84 (58-143)	0	26	0
**Colic_15**	Colonic sand impaction	0*	18	70 (36-143)	0	6	0
**Colic_16**	Nephrosplenic entrapment and colonic impaction	1	24	46 (32-145)	0	3	0
**Colic_17**	Nephrosplenic entrapment and colonic impaction	1	24	47 (28-144)	0	6	5
**Colic_18**	Colonic impaction	1	4	53 (30-110)	0	2	0
**Colic_19**	Unspecific	1	24	51 (36-171)	0	1	0
**Colic_20**	Colonic impaction	1	8	47 (28-126)	0	6	0
**Colic_21**	Colonic impaction	1	24	42 (26-161)	16	34	4
**Colic_22**	Colonic impaction	0*	24	44 (28-199)	0	27	0

ECG = Electrocardiography, AV/SA block = Second degree AV and SA block, SVPC = Supraventricular premature complexes, VPC = Ventricular premature complexes.*Horses euthanized had the diagnosis confirmed by necroscopy. **Three episodes of ventricular tachycardia (VT) observed.

Diagnoses, survival, duration of ECG recording in hours, mean HR (range), and numbers of arrhythmias. 1= Survived, 0= Euthanized.

### ECG analysis

The quality of the ECG recordings was evaluated as good, and the hours listed in Table 1 were all acceptable and readable with clear QRS complexes.

### Heart rate

**Figure 1:**
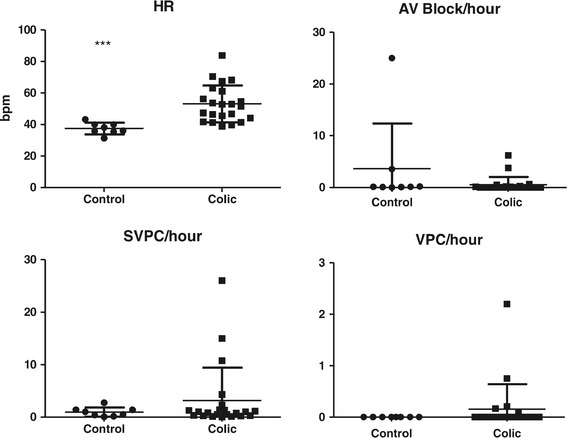
**Heart rate and distribution of arrhythmias in controls and colic horses.** Arrhythmias/hour, mean ± SD. Significant differences are indicated by asterisks (*** = P < 0.001).

The HR (mean ± SD) in the control group was 37.4 ± 3.7 bpm (range 31.2-43.3) and for the colic group 51.6 ± 11.8 bpm (range 38.8-83.9) (Figure 1). There was significant difference between the two group means (*P <* 0.001), see Table 1 for individual horses.

### Cardiac rhythm

Diagnosis, survival, ECG recording time, HR, and numbers of arrhythmias are presented in Table 1, and arrhythmias/hour is illustrated in Figure 1. As only four horses showed SA block (1-2 SA block per horse) these were included in the group of second degree AV block. Second degree AV blocks and SVPCs were found in both groups, whereas VPCs only were present in 5 colic horses. In one colic horse (colic_5) three episodes of VT were observed. The frequencies of AV block/hour were 3.6 ± 8.7 (mean ± SD), range 0.0-25.0 in the control group and 0.5 ± 1.5, range 0.0-6.2 in the colic group. The frequencies of SVPC/hour were 1.0 ± 0.9, range 0.1-2.8 in the control group and 3.2 ± 6.3, range 0.04-26.0 in the colic group. The frequency of VPC/hour in the colic group were 0.2 ± 0.5, range 0.0-2.2. There were no significant differences in the mean number of arrhythmias between groups, AV block (*P* = 0.08) and SVPC (*P* = 0.76). No horses in the control group had a VPC and a *t*-test could not be performed. Instead, a 95% confidence interval (CI) was calculated for the colic group (95%CI = -0.06 – 0.37). Since zero was included in the 95% CI there was no significant difference between the colic and the control group. Using linear regression, we tested if the electrolyte concentration at admission (T = 0) could explain the mean number of arrhythmias per hours for the horses in the following 24 hours. There was no significant effect on the frequency of SVPC by any of the electrolytes (potassium, sodium, ionized calcium, chloride and total magnesium) or the metabolites glucose and L-lactate. Comparing VPC and the blood parameters, a significant linear association for sodium (*P* = 0.045) and chloride (*P* = 0.043) was found. However, as one horse (Colic_5) had extreme values with high numbers of VPCs (Table 1), the calculations were repeated omitting this horse, which resulted in no significant linear association between VPCs regressed on either sodium (*P* = 0.20) or chloride (*P* = 0.73). Furthermore, the coefficients of determination (r^2^) were low in all regressions (r^2^ < 0.14).

### Electrolyte analysis

Descriptive values (mean, SD, and range) for both groups at T = 0 and T = 24 for the blood parameters are shown in Table 2. All of the mean values of control horses (n=8) were within the reference range. However, one horse had elevated potassium level at T=0 and one horse had reduced potassium level at T=0 and elevated ionized calcium level in both blood samples. For the colic horses, the mean values of total magnesium and chloride were below the reference range, while the mean of glucose and L-lactate were above the reference range at admission (T = 0). At T = 0 a large number of colic horses had blood parameters outside the reference values stated by the laboratory, i.e. 16 out of 22 horses had glucose higher than reference values and for 13 of the colic horses total magnesium was below reference values. See numbers of horses outside reference values in Table 2.

**Table 2:** **Blood parameters at admission (T = 0) and 12-24 hours after (T = 24) in control (n = 8) and colic (n = 22) horses**

	**Lab reference value**	**Control (n = 8)**								**Colic (n = 22)**							
		**T = 0**				**T = 24**				**T = 0**				**T = 24†**			
	**Range**	**Mean**	**SD**	**Range**	**# horses**	**Mean**	**SD**	**Range**	**# horses**	**Mean**	**SD**	**Range**	**# horses**	**Mean**	**SD**	**Range**	**# horses**
**K** ^**+**^ **(mmol/L)**	3.0-5.0	4.3	0.38	3.9-5.1	1 (>)	3.6	0.62	2.2-4.1	1 (<)	3.4	0.38	2.6-4.3	1 (<)	3.3	0.92	2.8-3.6	2 (<)
**Na** ^**+**^ **(mmol/L)**	132-146	138	1.05	136-139	0	139	1.51	136-141	0	136	2.70	130-139	2 (<)	137	35.43	136-140	0
**iCa** ^**2+**^ **(mmol/L)**	1.4-1.7	1.7	0.04	1.6-1.8	1 (>)	1.7	0.07	1.5-1.8	1 (>)	1.4	0.13	1.2-1.7	8 (<)	1.5	0.41	1.4-1.7	0
**tMg** ^**2+**^ **(mmol/L)**	0.74-1.20	NA	NA	NA	0	NA	NA	NA	0	0.71*	0.27*	0.49-1.71*	13 (<)	NA	NA	NA	NA
**Cl** ^**-**^ **(mmol/L)**	98-110	103	1.94	99-106	0	102	2.10	98-104	0	97	3.26	90-104	11 (<)	102	26.48	95-105	1 (<)
**Glucose (mmol/L)**	4.2-6.4	5.2	0.29	4.7-5.6	0	5.1	0.48	4.3-5.7	0	8.4	2.36	4.9-12.3	16 (>)	7.3	2.78	5-13.3	9 (>)
**L-Lactate (mmol/L)**	< 1.5	0.7	0.16	0.5-0.9	0	0.5	0.15	0.3-0.7	0	3.2	3.58	0.6-16	14 (>)	2.1	3.18	0.6-12.4	3 (>)
**PCV (%)**	32-50	35	2.76	32-40	0	37	4.91	31-47	0	39	7.75	24-60	1 (>)	34	6.08	27-46	0
**pH**	7.32-7.46	7.37	0.02	7.35-7.40	0	7.38	0.02	7.36-7.42	0	7.38	0.05	7.23-7.45	2 (<)	7.36	0.07	7.30-7.41	2 (<)

Mean, ± SD. Range for potassium (K^+^), sodium (Na^+^), ionized calcium (iCa^2+^), total magnesium (tMg^2+^), chloride (Cl^-^), glucose, and L-lactate. # horses denotes the number of horses that are outside the reference value. The brackets (<) or (>) indicate whether the values are below or above the reference values.

*SD* standard deviation, *n = 17, † n = 14, *NA* not available.

**Figure 2:**
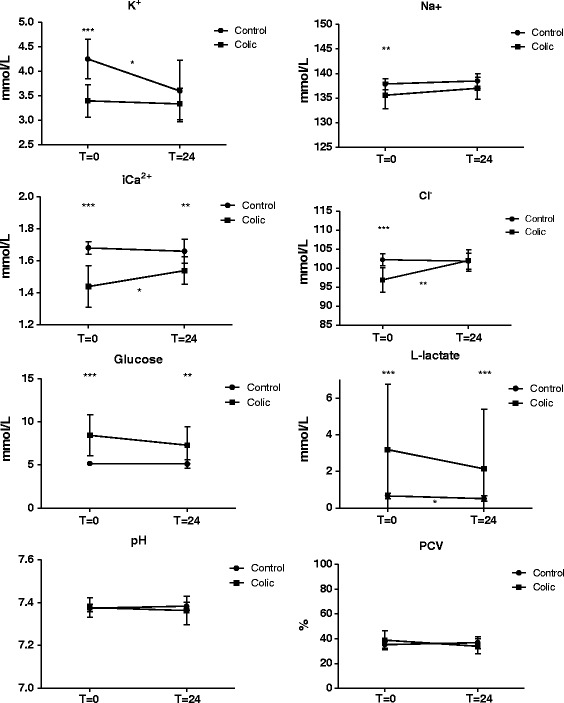
**Distribution of blood parameters.** Mean ± SD for both groups at T = 0 and T = 24. Significant differences are indicated by asterisks (* = P < 0.05, ** = P < 0.01, *** = P < 0.001). The asterisks placed above the vertical bars indicate significant difference between the two groups. The asterisk placed nearby the horizontal bars indicate significant difference between T = 0 and T = 24 within the group (either control or colic).

Comparison of means between control horses and colic horses at T = 0 showed significant differences in means of potassium (*P <* 0.001), sodium (*P* = 0.003), ionised calcium (*P* < 0.001), chloride (*P* < 0.001), glucose (*P* < 0.001), and L-lactate (*P* < 0.001). At the time of the second blood sample (T = 24), a significant difference was still present for ionised calcium (*P* = 0.003), glucose (*P* = 0.0026), and L-lactate (*P* < 0.001). No statistical significant difference in pH and PCV was found (Figure 2).

Repeated measurements were analysed within the controls and colic groups comparing T = 0 and T = 24. A significant difference was found between start (T = 0) and end (T = 24) measurements in control horses for potassium (*P* = 0.022) and L-lactate (*P* = 0.014). For the colic horses, a significant increase in ionised calcium (*P* = 0.042) and chloride (*P* = 0.005) was found, whereas no significant changes for sodium and magnesium were observed. There was no significant difference in means of PCV or pH within or between groups at any time. Mean and SD are illustrated for all electrolytes, the metabolites glucose and L-lactate, pH, and PCV in Figure 2.

## Discussion

This study describes the number of cardiac arrhythmias in colic horses compared to controls, and the association between arrhythmias and electrolyte disturbances.

Physiological arrhythmias are common in clinically normal horses at rest, such as sinus arrhythmias, second degree AV blocks and SA blocks [13,14]. The majority of physiological arrhythmias are believed to be related to fluctuations in autonomic tone as horses exhibit high vagal tone, and it is well known that heart rate variability is positively correlated with parasympathetic activity within HR interval of 20-100 bpm [15-17]. In the present study, seven out of eight control horses exhibited AV blocks while this arrhythmia was present in eight out of 22 colic horses. Due to the stressful and painful condition most of the colic horses experienced, we would not expect them to develop AV blocks as theses arrhythmias primarily occur in resting clinically normal horses, probably influenced by the predominant parasympathetic nervous system. Nevertheless, a relatively high number of the colic horses had AV blocks, and we could therefore not document a significant difference between the two groups. One explanation for this could be that most of the colic horses received α_2_-adrenergic agonists as part of the medical treatment, which induces SA and AV blocks [18]. Administration of these drugs could be the reason why a relatively high prevalence of colic horses had normal physiological arrhythmias, despite the elevated HR caused by pain and stress that would normally depress vagal tone. In the present study, the exact time of drug administration to the colic horses was not registered, and therefore we are not able to correlate AV blocks with medication.

Supraventricular premature complexes (SVPCs) were observed in both control and colic horses. This arrhythmia is frequently experienced in clinically normal horses during and after exercise [1-3], whereas most ventricular arrhythmias occur after exercise [1-3,19]. In post-operative colic horses and horses operated for elective orthopaedic diseases, high numbers of both supraventricular and ventricular premature contractions have been observed [6]. We did not observe a significant difference between numbers of SVPCs and VPCs between colic horses and control horses, yet only colic horses developed VPCs in our study. The numbers were decreased compared to a study in postoperative colic horses and post-operative orthopaedic cases [6] and in endotoxemic horses [20] suggesting that effect of anaesthesia and endotoxaemia on the myocardium is more arrhythmogenic compared to the colic disease by itself. However, it was not known whether these horses had VPCs prior to developing colic. One horse (colic_5), a 16-years-old Icelandic gelding diagnosed with peritonitis and severe typhlocolitis developed 22 VPCs during 10 hours of ECG. Compared to the other colic horses in the present study, 22 VPCs was remarkably high and this horse was showing severe pain and exhibited obvious signs of endotoxemia (cyanotic mucous membranes, elevated temperature, prolonged capillary refill time, severe leukopenia) before euthanasia was recommended to the owner. Disseminated necrotic typhlocolitis and peritonitis was diagnosed post mortem, and *Clostridium difficile* was cultured from faeces.

The maximum accepted deviation of RR-interval was set to 20% according to a study showing that normal RR variation were within 20% in resting horses with HR below 60 bpm. When HR increased, the variation between heart beats was reduced to 10% and when HR was above 100 bpm the beat-to-beat variation was within 4% (Flethøj *et al*. unpublished observations 2014). The mean HR for colic horses in the present study was 51.6 bpm, which was significantly higher than the controls having a mean HR of 37.4 bpm. It is therefore most likely that we have analysed parts of the colic horses using a too high variation percentage (20%) indicating that we might have underestimated the numbers of SVPCs in the colic horses. In a previous study of postoperative horses, high HR was identified as a risk factor for developing VPCs, but no explanation for this could be found [6].

In the colic group, levels of potassium, calcium, magnesium, sodium, and chloride were in the lower part of or below the reference range in the present study. While the decreased levels of potassium, calcium, and magnesium are consistent with previous studies [21-26], the decreased levels of sodium and chloride differ from previous studies reporting no significant difference between control and colic horses [25,26]. It has however been suggested that mild hyponatremia is a normal finding in colic horses [27]. The significant difference found for sodium between control and colic horses most likely has no clinical significance as only two horses had levels below reference range. The deficits of potassium, sodium, calcium, and magnesium can be caused by anorexia, retention of contents in the intestines, or loss into the intestinal lumen, all of which can be caused by colic [23,27]. Deficit of chloride can be caused by removal of hydrogen chloride from the ventricle via gastric decompression [28], which is performed regularly both before admission, and during hospitalization of all colic horses at the Large Animal Teaching Hospital. This could explain why 50% of the colic horses were hypochloremic at admission.

Significant difference in means between control and colic horses was shown for potassium, but beside one hypokalaemic colic horse all were within reference range, correlating with results reported previously [24,25]. Blood potassium levels vary with for example feed content and loss through sweat [27]. Hypokalaemia is associated with cardiac arrhythmias in equine case reports, but levels below 2.5 mmol/l are required [11] and in human patients cardiac arrhythmias are rare, even in severe hypokalemia [29].

Hypocalcaemia was only found in 30% of the colic horses in the present study, while previous studies have reported frequencies as high as 86% [23] and 100% [21] of post-operative colic horses. However, the previous studies were all performed in horses undergoing colic surgery. Since the cases presented here were not surgical cases, the relatively low prevalence of horses with hypocalcaemia reflects less severe cases, and the fact that they have not undergone an abdominal surgical procedure. It has been reported that hypocalcaemia may result in morphological electrocardiographic changes with increased QT interval suggesting prolonged repolarization. Prolonged repolarization predisposes for development of severe ventricular arrhythmias [23]. Based on current literature, hypocalcaemia is probably not solely responsible for detected arrhythmias, because no significant difference was found for ionised calcium between horses with and without arrhythmias in a study including hypocalcaemic horses [4].

The present study measured total magnesium concentration in the colic horses, and 77% of the horses were hypomagnesaemic at admission. A previous study reported 62.5% of colic horses having low total magnesium [24], while another study reported 17% of surgically managed colic horses to have low total magnesium [23]. The high prevalence in the present study might be explained by the small study population and inclusion of milder colic cases. The measurement of total magnesium may however have limited clinical relevance, as some colic horses are presented with metabolic acidosis [22,26], which inhibits the protein binding of magnesium, and thereby leaves the ionized fraction within normal limits. Measurement of ionised magnesium was not possible in the present study. Cardiac arrhythmias including SVPCs and VPCs have been observed in hypomagnesaemic horses [11], but those cases had much lower total blood magnesium than the horses included in the present study.

The hypokalaemia and hypomagnesaemia found in the present study could be a result of elevated insulin concentrations in the blood caused by hyperglycaemia and insulin resistance, as reported in critically ill humans and horses [30,31]. Insulin causes a shift of potassium and magnesium into the intracellular space, thereby decreasing the concentration in the blood [24]. The colic horses in the present study had significantly increased glucose levels in the blood and could be insulin resistant. However, we did not examine this further, nor did we treat the animals with insulin so this remains speculations.

In the present study, linear regression showed a significant association between low concentration of sodium, chloride, and increased numbers of VPCs. However, one horse had 22 VPCs during the 10 hours ECG recordings which had a great impact on the data, and when excluding this animal there was no significant association. In addition, r^2^ values were close to zero, which further indicates that the association should be questioned. Also, neither hyponatremia nor hypochloremia are recognised as correlated to cardiac arrhythmias [9,10]. Overall, the present study is therefore not able to document any significant associations between electrolyte disturbances and cardiac arrhythmias. In common with other equine hospitals, our patients receive intravenous electrolyte supplementations soon after admission correcting any profound electrolyte abnormalities, which may be the explanation why we did not observe cardiac arrhythmias associated with electrolyte disturbances. Some of the colic horses included in the present study were likely to be endotoxaemic, which is another factor that could contribute to the electrolyte disturbances observed. Additionally, as we did not register arrhythmias for each specific hour but calculated mean/hour we may miss information regarding arrhythmia distribution from admission to end of ECG recording.

Limitations in the current study include the relatively small population of horses, both control and colic horses. As the group of horses consisted of several colic diagnoses, the uniformity of the group was reduced. Relatively mild cases of colic horses were primarily included, but some lethal and endotoxaemic cases were also encountered. An additional limitation was the lack of standardised registration of treatment and drugs used in the colic horses during the ECG recording period, where α2- adrenergic agonists, metamizole natrium, and butorphanol, and various electrolytes enriched intravenous fluids are standardised treatment in the hospital.

## Conclusions

This study showed that referred colic horses developed AV blocks, SVPCs and VPCs during ECG measurements, but no significant difference was found between colic and control horses. Also, no significant correlation between electrolyte abnormalities and cardiac arrhythmias was found, and generally the changes in electrolytes, glucose, and L-lactate in the colic horses were consistent with the literature. This study indicates that colic per se is not pro-arrhythmogenic, whereas more systemically severe diseases such as endotoxaemia probably are more likely to result in ventricular arrhythmia. Larger scale studies including multi-center studies are required to identify risk factors for development of cardiac arrhythmias in this group of equine patients.
